# High flow nasal cannula oxygen therapy versus non-invasive ventilation for acute exacerbations of chronic obstructive pulmonary disease with acute-moderate hypercapnic respiratory failure: a randomized controlled non-inferiority trial

**DOI:** 10.1186/s13054-024-05040-9

**Published:** 2024-07-18

**Authors:** Dingyu Tan, Bingxia Wang, Peng Cao, Yunyun Wang, Jiayan Sun, Ping Geng, Joseph Harold Walline, Yachao Wang, Chenlong Wang

**Affiliations:** 1 Department of Emergency, Northern Jiangsu People’s Hospital Affiliated to Yangzhou University, Yangzhou, 225001 China; 2 Pharmacy Department, Northern Jiangsu People’s Hospital Affiliated to Yangzhou University, Yangzhou, 225001 China; 3 Department of Emergency Medicine, Penn State Health Milton S. Hershey Medical Center, 500 University Drive, Hershey, PA 17033 USA

**Keywords:** Chronic obstructive pulmonary diseases, Respiratory failure, High-flow nasal cannula oxygen therapy, Non-invasive ventilation, Randomized controlled trial

## Abstract

**Background:**

Although cumulative studies have demonstrated a beneficial effect of high-flow nasal cannula oxygen (HFNC) in acute hypercapnic respiratory failure, randomized trials to compare HFNC with non-invasive ventilation (NIV) as initial treatment in acute exacerbations of chronic obstructive pulmonary disease (AECOPD) patients with acute-moderate hypercapnic respiratory failure are limited. The aim of this randomized, open label, non-inferiority trial was to compare treatment failure rates between HFNC and NIV in such patients.

**Methods:**

Patients diagnosed with AECOPD with a baseline arterial blood gas pH between 7.25 and 7.35 and PaCO_2_ ≥ 50 mmHg admitted to two intensive care units (ICUs) at a large tertiary academic teaching hospital between March 2018 and December 2022 were randomly assigned to HFNC or NIV. The primary endpoint was the rate of treatment failure, defined as endotracheal intubation or a switch to the other study treatment modality. Secondary endpoints were rates of intubation or treatment change, blood gas values, vital signs at one, 12, and 48 h, 28-day mortality, as well as ICU and hospital lengths of stay.

**Results:**

225 total patients (113 in the HFNC group and 112 in the NIV group) were included in the intention-to-treat analysis. The failure rate of the HFNC group was 25.7%, while the NIV group was 14.3%. The failure rate risk difference between the two groups was 11.38% (95% CI 0.25–21.20, *P* = 0.033), which was higher than the non-inferiority cut-off of 9%. In the per-protocol analysis, treatment failure occurred in 28 of 110 patients (25.5%) in the HFNC group and 15 of 109 patients (13.8%) in the NIV group (risk difference, 11.69%; 95% CI 0.48–22.60). The intubation rate in the HFNC group was higher than in the NIV group (14.2% vs 5.4%, *P* = 0.026). The treatment switch rate, ICU and hospital length of stay or 28-day mortality in the HFNC group were not statistically different from the NIV group (all *P* > 0.05).

**Conclusion:**

HFNC was not shown to be non-inferior to NIV and resulted in a higher incidence of treatment failure than NIV when used as the initial respiratory support for AECOPD patients with acute-moderate hypercapnic respiratory failure.

*Trial registration*: chictr.org (ChiCTR1800014553). Registered 21 January 2018, http://www.chictr.org.cn

## Introduction

Chronic obstructive pulmonary diseases (COPD) is a common chronic respiratory disease which continues to have high morbidity and mortality worldwide. Acute exacerbations of COPD (AECOPD) are the leading cause of death in COPD patients, and non-invasive ventilation (NIV) is recommended as standard therapy for AECOPD with moderate hypercapnic acute respiratory failure (ARF) [1]. However, several factors may affect the treatment outcome for NIV, such as the severity of the disease, patient-ventilator interaction, discomfort related to the mask interface, and the skill of the medical team in managing NIV [2, 3].

High-flow nasal cannula oxygen (HFNC) has been shown to have beneficial effects for stable COPD patients [4]. High-flow gas can generate a positive airway pressure that may counterbalance intrinsic positive end-expiratory pressure. Additionally, HFNC can cause a washout effect of the nasopharyngeal dead space to improve ventilatory efficiency and carbon dioxide removal [5]. The warmed and humidified gas can prevent bronchoconstriction in response to otherwise dry air, enhance mucociliary clearance, and decrease inspiratory resistance and diaphragmatic effort [6]. In recent years, the application of HFNC in AECOPD or hypercapnic ARF patients has steadily increased. In a study which enrolled 38 AECOPD patients with pH < 7.38, HFNC was reported to increase pH by 0.052 and decrease the partial pressure of arterial carbon dioxide (PaCO_2_) by 9.1 mmHg, while also showing that the effect of HFNC was more obvious in patients with pH < 7.35 [7]. Additionally, HFNC was shown to improve patients’ pH and respiratory rate (RR) among 30 patients with moderate hypercapnic ARF who were intolerant to NIV, while the non-response rate was only 13.3% [8]. In two observational studies with larger samples, HFNC was reported to have similar treatment failure rates as NIV in AECOPD patients with moderate hypercapnic ARF, while HFNC had better patient tolerance [9, 10]. The efficacy of HFNC in the treatment of AECOPD needs to be further confirmed by randomized controlled trials (RCTs).

Non-inferiority design has been used in the comparison between HFNC and NIV, however, non-inferiority RCTs comparing treatment failure of HFNC and NIV in AECOPD have so far been rare. We hypothesized that HFNC and NIV had similar treatment failure rates for AECOPD patients with moderate hypercapnic ARF.

## Materials and methods

### Study design and ethics approval

This was a single center, non-inferiority, unblinded RCT, registered at chictr.org (ChiCTR1800014553). The study was performed in the respiratory intensive care unit (RICU) and the emergency intensive care unit (EICU) of an urban, tertiary care, university hospital in China from March 2018 to December 2022. This study was approved by the hospital’s Institutional Ethics Committee (No. 2017053) and conformed to the Helsinki Declaration guidelines and medical research ethics standards. Informed consent was obtained from all enrolled patients or their relatives.

### Patient screening

AECOPD patients with moderate hypercapnic ARF were screened for enrollment. The diagnosis of AECOPD (any worsening of respiratory symptoms that was beyond normal day-to-day variation and led to changes in medication in suspected or confirmed COPD patients) was established using the 2017 GOLD criteria [11]. Moderate hypercapnic ARF was defined as respiratory acidosis with a blood gas pH range between 7.25 and 7.35 and a PaCO_2_ ≥ 50 mmHg. Exclusion criteria were: age < 18 years old, anyone requiring immediate endotracheal intubation (e.g. those with severe hypoxia such as the ratio of partial pressure of arterial oxygen (PaO_2_) / the fraction of inspiration oxygen (FiO_2_) < 150 mmHg without external positive airway pressure, severe respiratory acidosis with a pH < 7.25, RR ≥ 40 breaths/min, or a Glasgow coma score < 8), contraindications to NIV or HFNC (poor sputum excretion ability, oral or facial trauma, significant hemodynamic instability), poor short-term prognosis (those already receiving palliative care or at very high risk of death within seven days due to existing medical/surgical pathology), presence of a tracheostomy, other organ failure, or patients (or their relatives) who could not give informed consent. Patients who withdrew informed consent were secondarily excluded.

### Experimental procedure

Patients were divided into HFNC and NIV groups by computer-generated random number sequencing. Using opaque envelopes for covert distribution, with ten envelopes per group, five HFNC and five NIV, so that the number of patients in the two groups were evenly distributed.

For patients in the NIV group, a dedicated NIV (Philips V60 or BiPap Vision) with a standard oral-nasal mask (RT040) were used in S/T mode. The initial NIV settings were as follows: expiratory positive airway pressure was set to 4 cmH_2_O, the inspiratory positive airway pressure was set to 8 cmH_2_O, and the both pressures were gradually increased judged to acceptable tolerance by the patient. The expiratory positive airway pressure, inspiratory positive airway pressure and FiO_2_ were adjusted under the attending physician’s instruction to maintain a 6–8 ml/kg ideal body weight tidal volume, a pulse oxygen saturation (SpO_2_) of 88–92%, and a RR ≤ 28/min with appropriate inspiratory triggering and effort. The initial use of NIV was targeted to last at least two hours and then continued as needed. NIV could be used intermittently based on patient tolerance. During the intermittent period of NIV, a traditional (low-flow) nasal cannula oxygen could be used. Treatment time was gradually reduced in patients whose blood gas and other respiratory indices sufficiently improved. NIV was discontinued when the total daily treatment duration was less than four hours after the patient's clinical and blood gas values improved past certain cut-offs (pH > 7.35 and PaCO_2_ < 45 mmHg or > 45 mmHg with RR < 25 breaths/min). NIV could be restarted in case of clinical or blood gas value worsening.

In the HFNC group (AIRVO™ 2, Fisher & Paykel Healthcare, Auckland, New Zealand), subjects received an initial airflow of 40 L/min at a temperature of 37 °C through suitable nasal prongs. The FiO_2_ was adjusted to maintain a SpO_2_ between 88 and 92%. The airflow and temperature were adjusted according to patient tolerance. If patients in HFNC group tolerated the apparatus well, the treatment was continued, or it could be applied intermittently. Oxygen therapy during the intermittent period of HFNC was otherwise the same as in the NIV group. FiO_2_ was gradually reduced to < 35% at first in patients with stable clinical status and blood gas. If the patient had no obvious respiratory distress with a stable PaCO_2_, airflow was gradually reduced in a step-wise rate of 5–10 L/min per reduction. HFNC was discontinued when the airflow was reduced to 15L/min or less for more than two hours, and could be restarted if clinical status and blood gas deteriorated.

During treatment, if the patient could not tolerate the assigned treatment, or had respiratory distress, hypoxia, or carbon dioxide retention unalleviated by assigned treatment, the patient would be changed to the other study treatment modality. These switches were decided by the patient’s attending physician. The patient’s group classification would not be changed as a result of such a switch in treatment modality (intention to treat analysis), but would be recorded for statistical analysis.

The criteria for invasive mechanical ventilation in our study were: progressively increasing PaCO_2_ with pH ≤ 7.20, severe hypoxia (defined by as a PaO_2_ < 50 mmHg despite FiO_2_ > 0.5), RR > 40 breaths or < 8 breaths per minute, inability to protect their airway, or respiratory or cardiac arrest [11].

### Data collection

Sex, age, relevant comorbidities, COPD duration (in years), any respiratory medications, recent pulmonary function tests, time of ICU admission, and severity score including the acute physiological and chronic health status score II (APACHE II), and the Simplified Acute Physiology Score II (SAPS II) for eligible patients were all recorded. Vital signs (heart rate, blood pressure, RR, and SpO_2_) as well as arterial blood gas analysis results were recorded when entering the ICU (baseline state), and again after one hour, 12 h, then once a day thereafter. The collection of vital signs and blood gas analysis results was stopped if the patient received invasive ventilation.

The initial settings of NIV or HFNC, daily total respiratory support time, and any changes in respiratory support modality (changes from NIV to HFNC or from HFNC to NIV, or a change to invasive ventilation, including specific time and reasons for such changes) were also collected. We recorded the daily total number of nursing airway care interventions (such as correcting unplanned device displacement, assisting in spitting, eating, etc.), Borg dyspnea score, comfort (visual analog scale) score, and adverse reactions to treatment (e.g. excessive air flow, eye irritation, epistaxis, abdominal distension, claustrophobic feelings, or skin breakdown). The 28-day survival of the patients was determined according to electronic medical and follow-up records.

### Endpoints

The primary endpoint was treatment failure, defined as invasive ventilation or a switch in respiratory treatment modality. Secondary endpoints included invasive ventilation, treatment switch, vital signs (RR, heart rate, and blood pressure) and arterial blood gas analysis results (pH, PaCO_2_, and PaO_2_/FiO_2_) at one, 12, and 48 h, as well as 28-day mortality, and ICU and hospital lengths of stay. The number of nursing airway care interventions within the first 24 h, Borg dyspnea score and comfort score after 12 h of treatment, the duration of respiratory support, treatment intolerance and the incidence of adverse effects were also analyzed. The definition of nursing airway care interventions, the evaluation method of dyspnea score and comfort score were previously described in additional detail [12].

### Statistical analysis

According to previous studies, it was estimated that NIV would fail in 22% of patients with COPD [1]. The absolute difference in treatment failure rates between HFNC and NIV was predicted to fall within a range of 4–12% [12, 13]. In this study, we set the non-inferiority cutoff at 9% after discussions with two senior pulmonologists and one critical care specialist. To assess non-inferiority using an α = 0.05, β = 0.20, and a dropout rate of 5%, each group was estimated to need at least 114 subjects.

The analysis of the main endpoint was performed on both an intention-to-treat and a per-protocol basis. Secondary endpoints were analyzed on an intention-to-treat basis. Kaplan–Meier curves with the log rank test were used to assess patient survival and time to treatment failure. Categorical variables were expressed as frequencies and percentages, using χ^2^ test or Fisher’s exact probability tests. Continuous data were expressed as means ± standard deviation or medians with interquartile (25–75th) percentiles, and were analyzed with Student’s t-test or the rank-sum test. Repeated measures analysis of variance, or non-parametric tests of multiple correlated samples (Friedman test for heterogeneity of variance or the skewed distributed data) followed by Bonferroni’s test were performed for the data obtained at multiple time points. *P*-values < 0.05 were considered statistically significant. SPSS 26.0 (IBM Corporation, Armonk, NY, USA) was used for all data analysis.

## Results

### Patient characteristics

**Fig. 1:**
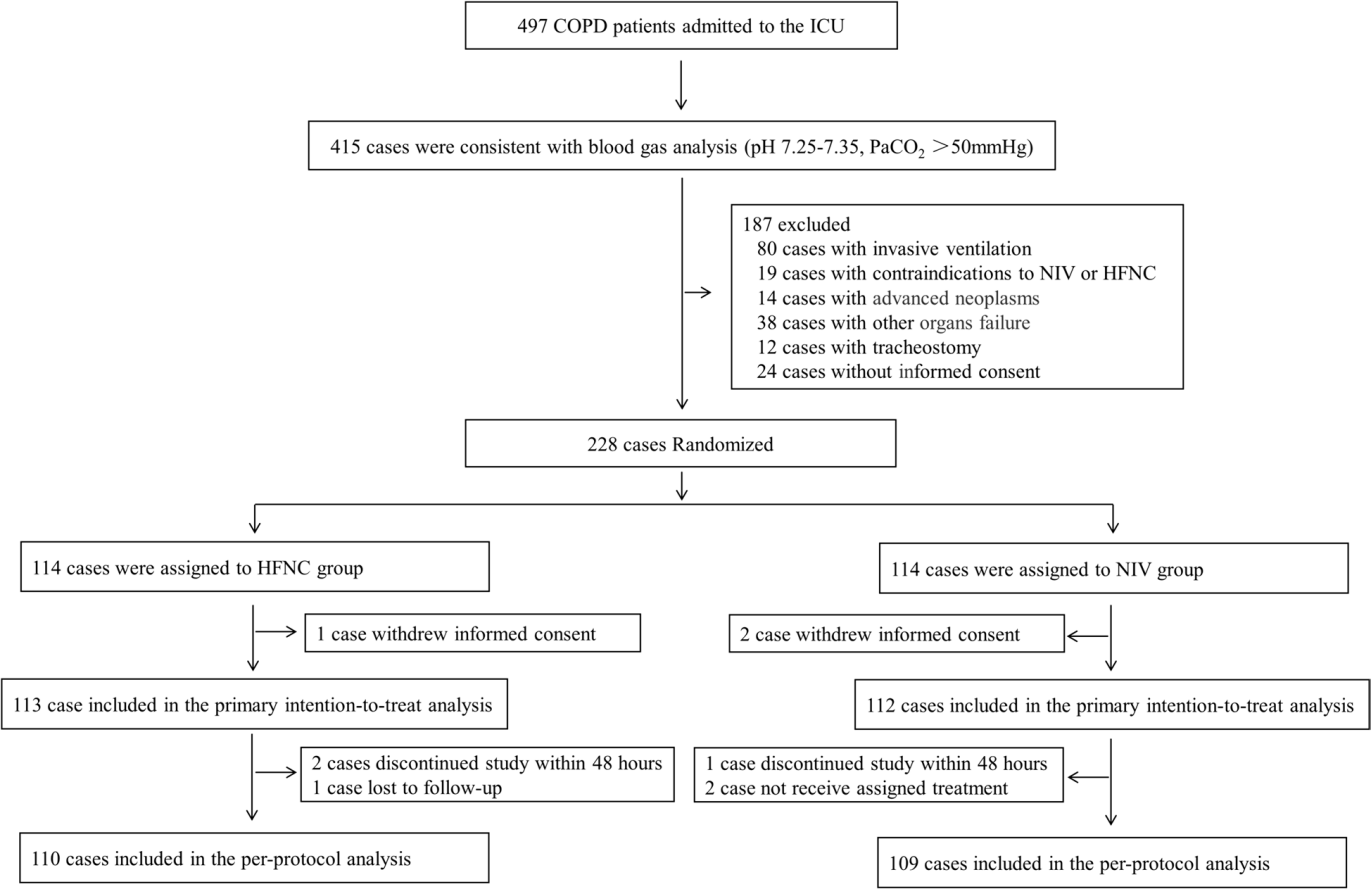
Flow chart of patient enrollment. COPD: Chronic obstructive pulmonary disease; ICU: Intensive care unit; HFNC: High-flow nasal cannula oxygen therapy; NIV: Non-invasive ventilation

497 COPD patients were admitted to our two ICU units during the study period. 415 patients met the blood gas analysis criteria of hypercapnic ARF in AECOPD. Among these 415 patients, 228 patients were randomized to the HFNC or NIV groups after 187 subjects were excluded for various reasons (see Fig. 1). Three patients withdrew informed consent and were secondarily excluded. Finally, 113 patients in the HFNC group and 112 patients in the NIV group were included in the intention-to-treat analysis. In the two groups, demographic, COPD duration, smoking history, relevant comorbidities, COPD medications, respiratory therapy at home, and pulmonary function tests were similar (all *P* > 0.05, see Table 1). There were also no significant differences in APACHE II scores, SAPS II scores, vital signs or arterial blood gas analysis results between the two groups (all *P* > 0.05).

**Table 1:** Baseline characteristics of selected patients

Characteristics		HFNC (n = 113)	NIV (n = 112)	*P* value
Male, n (%)	71 (62.8)		62 (55.4)	0.254
Age, years	73 (65–78)		69 (63–76)	0.082
History of COPD, years	8 (6–12)		8 (6–11)	0.490
Smoking history, n (%)				
Current	12 (10.6)		7 (6.3)	0.239
Former smoker	36 (31.9)		49 (43.8)	0.066
Comorbidities, n (%)				
Diabetes mellitus	25 (21.1)		31 (27.7)	0.335
Coronary artery disease	49 (43.4)		38 (33.9)	0.146
Chronic liver disease	9 (8.0)		15 (13.4)	0.187
Chronic kidney disease	24 (21.2)		15 (13.4)	0.120
Cerebrovascular disease	11 (9.7)		19 (17.0)	0.111
Malignancy	13(11.5)		16 (14.3)	0.534
Medication before exacerbation, n (%)				
Inhaled corticosteroids	21 (18.6)		33 (29.5)	0.056
Beta adrenoceptor agonist	50 (44.2)		44 (39.3)	0.451
Anticholinergics	23 (20.4)		32 (28.6)	0.152
Home oxygen therapy, n (%)				
NCO	23 (20.4)		18 (16.1)	0.405
NIV	9 (8.0)		12(10.7)	0.478
Pulmonary function class, n (%)	35		44	
II	14 (40.0)		17 (38.6)	0.902
III	19 (54.3)		24 (54.5)	0.982
IV	2 (5.7)		3 (6.8)	0.841
Mean length from acute attack to ICU admission, days	5 (3–8)		4 (3–7)	0.118
On admission to ICU				
APACHE II score	14 (11–17)		12 (10–16)	0.067
SAPS II score	32 (26–37)		29 (26–34)	0.201
Heart rate, beats/min	92 (85–101)		96 (85–103)	0.148
Respiratory frequency, /min	28 (25–30)		29 (26–32)	0.061
Mean arterial pressure, mmHg	88 (82–93)		84 (77–93)	0.090
Arterial pH	7.31(7.29–7.33)		7.30(7.28–7.32)	0.342
PaCO_2_, mmHg	63 (59–68)		61 (58–65)	0.130
PaO_2_/FiO_2_, mmHg	175 (167–199)		184 (167–202)	0.170

*HFNC* High flow nasal cannula; *NIV* Non-invasive ventilation; *COPD* Chronic obstructive pulmonary disease; *NCO* Nasal cannula oxygen; *ICU* Intensive care unit; *APACHE II*: Acute Physiology and Chronic Health Evaluation II; *SAPS II* Simplified acute physiology score II: *PaCO*_*2*_ Partial pressure of arterial carbon dioxide; *PaO*_*2*_ Partial pressure of arterial oxygen

### Primary endpoint and cause analysis

**Fig. 2:**
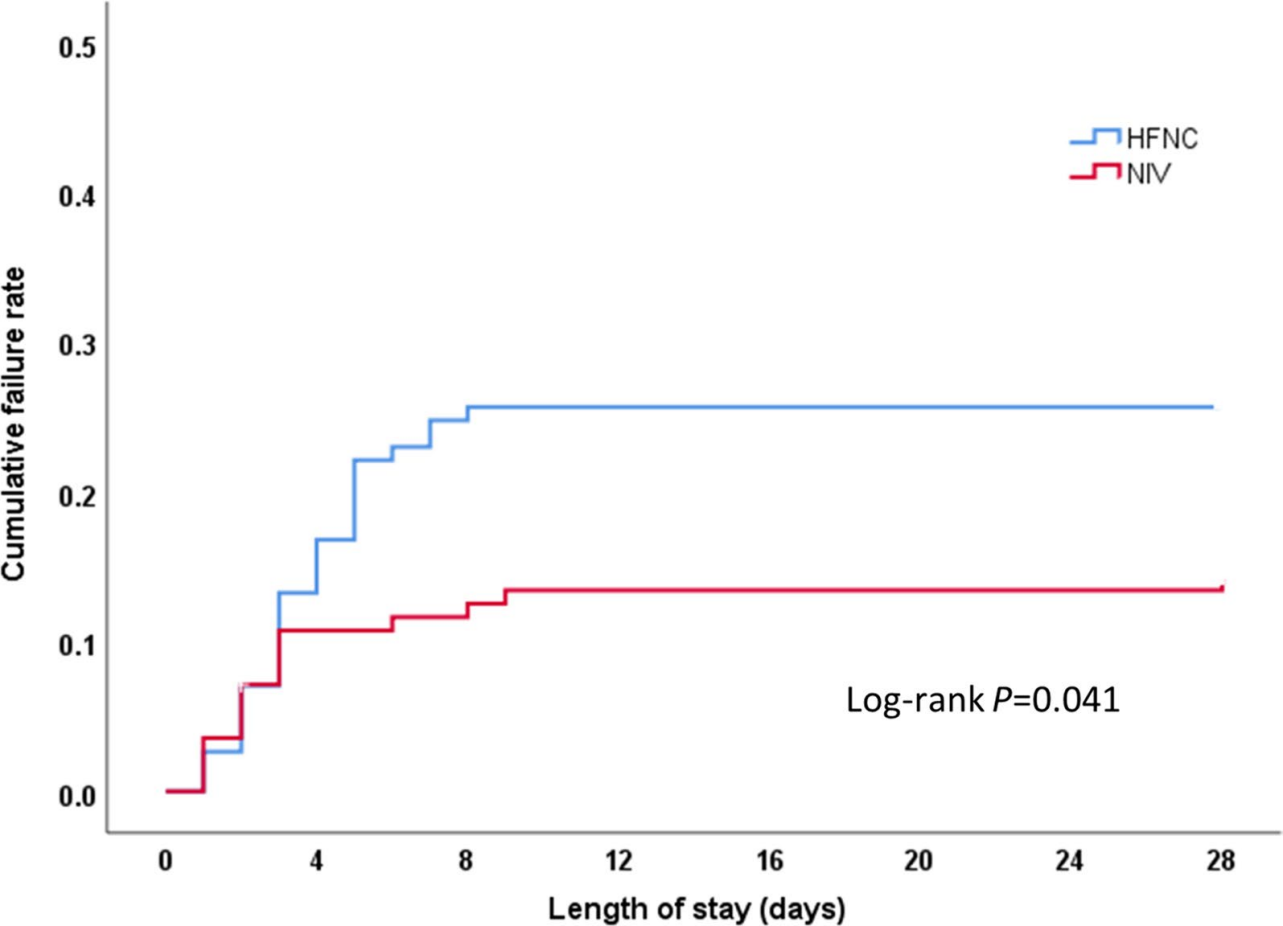
Kaplan–Meier curve analysis for cumulative failure rate. HFNC: High-flow nasal cannula oxygen therapy; NIV: Non-invasive ventilation

In the intention-to-treat analysis, the treatment failure rate in the HFNC group was 25.7% and 14.3% in the NIV group, with a difference between the two groups of 11.38% (95% CI 0.25–21.20, *P* = 0.033), which was higher than the non-inferiority threshold of 9%. Kaplan–Meier curve analysis showed that the cumulative failure rate of the HFNC group was significantly higher than that of the NIV group (Log Rank test 4.158, *P* = 0.041, see Fig. 2). In the per-protocol analysis, treatment failure occurred in 28 of the 110 HFNC patients (25.5%) and in 15 of the 109 NIV patients (13.8%) (risk difference, 11.69%; 95% CI 0.48–22.60).

Summarizing treatment failures in the HFNC group showed that the most common reasons for failure were carbon dioxide retention and exacerbations of respiratory distress, accounting for 44.8% and 31.0%, respectively. While in the NIV group, the most common reasons for failure were also carbon dioxide retention and exacerbations of respiratory distress, both accounting for 37.5%. (See Table 2).

**Table 2:** Primary endpoint and cause analysis

	HFNC	NIV	Risk difference, % (95% CI)	P value
Treatment failure, n (%)				
Intention-to-treat analysis	29/113 (25.7)	16/112 (14.3)	11.38 (0.25 ~ 21.20)	0.033
Per-protocol analysis	28/110 (25.5)	15/109 (13.8)	11.69 (0.48 ~ 22.60)	0.029
Analysis of treatment failure, n (%)				
Aggravation of respiratory distress	9/29 (31.0)	6/16 (37.5)	-6.47 (-37.06 ~ 22.64)	0.660
Aggravation of hypoxemia	7/29 (24.1)	4/16 (25.0)	-0.86 (-31.41 ~ 25.68)	0.949
Aggravation of carbon dioxide retention	13/29 (44.8)	6/16 (37.5)	7.33 (-24.74 ~ 35.94)	0.373

*HFNC* High flow nasal cannula oxygen therapy; *NIV* on-invasive ventilation

### Secondary endpoints

The rate of endotracheal intubation in the HFNC group was higher than that in the NIV group (14.2% vs 5.4%, *P* = 0.026). The treatment switch rate in the HFNC group was 11.5%, which was not statistically different from the NIV group (8.9%, *P* = 0.505) (see Table 3).

**Table 3:** Secondary endpoints in the HFNC and NIV groups

	HFNC (n = 113)	NIV (n = 112)	*p* value
Invasive ventilation	16 (14.2)	6 (5.4)	0.026
Treatment switch	13 (11.5)	10 (8.9)	0.524
Length of stay in ICU, days	7 (6–9)	9 (6–11)	0.059
Length of stay in hospital, days	10 (8–13)	11 (9–13)	0.228
28-day mortality, n (%)	11 (9.7)	8 (7.1)	0.485

*HFNC* High-flow nasal cannula oxygen therapy; *NIV* Non-invasive ventilation; *ICU* Intensive care unit

The mean arterial pressure and heart rate after initiating treatment showed no significant difference between the two groups at different time points. In the dynamic observation during the first 48 h, the RR gradually decreased over time in both groups. The RR in the NIV group at 48 h was lower than that in the HFNC group [23 (20–26) breaths per min vs 23 (22–27) breaths per min, *P* = 0.025, see Table 4)]. However, there was no significant difference in RR between the two groups at one hour and 12 h.

**Table 4:** Vital signs and arterial blood gas analysis

Characteristics	Group		Baseline		1h		12h		48h		P* value
Heart rate (beats/min)	HFNC		92 (85–101)		88 (83–100)		89 (84–98)		90 (82–100)		0.210
	NIV		96 (85–103)		93 (83–104)		90 (83–102)		88 (79–99)^abc^		0.001
	*P^* value		0.148		0.183		0.582		0.329		0.115^#^
Mean arterial pressure (mmHg)	HFNC		88 (82–93)		86 (78–93)		84 (76–93)		87 (79–94)		0.102
	NIV		84 (77–93)		86 (77–95)		83 (75–92)		85 (78–93)		0.100
	*P^* value		0.090		0.754		0.713		0.296		0.558^#^
Respiratory rate (min)	HFNC		28 (25–30)		26 (23–30)		25 (22–28)^a^		23 (22–27)^a^		< 0.001
	NIV		29 (26–32)		25 (21–30)^a^		23 (21–27)^a^		23 (20–26)^abc^		< 0.001
	*P^* value		0.061		0.118		0.095		0.025		0.197^#^
Arterial pH	HFNC		7.31(7.29–7.33)		7.33(7.31–7.36)^a^		7.37(7.34–7.39)^ab^		7.39(7.35–7.42)^abc^		< 0.001
	NIV		7.30(7.28–7.32)		7.34(7.31–7.37)^a^		7.37(7.35–7.40)^ab^		7.39(7.37–7.42)^abc^		< 0.001
	*P^* value		0.342		0.105		0.071		0.060		0.037^#^
PaCO_2_ (mm Hg)	HFNC		63 (59–68)		61 (56–64)^a^		57 (53–64)^ab^		57 (52–60)^ab^		< 0.001
	NIV		61 (58–65)		58 (53–64)^a^		56 (50–63)^ab^		53 (49–59)^abc^		< 0.001
	*P^* value		0.130		0.067		0.054		0.043		0.012^#^
PaO_2_/FiO_2_ (mmHg)	HFNC		175 (167–199)		215 (196–242)^a^		206 (192–225)^ab^		225 (207–253)^abc^		< 0.001
	NIV		184 (167–202)		206 (189–232)^a^		217 (195–234)^ab^		216 (203–238)^ab^		< 0.001
	*P^* value		0.170		0.069		0.066		0.058		0.798^#^

P* for overall comparisons of differences in the same group over time. *P*^#^ for overall comparisons of differences between the two groups over time. *P^* for comparisons of differences between the two groups at the same time point. ^a^Compared with the baseline value in the same group, *P* < 0.05 after Bonferroni correction. ^b^Compared with the 1h value in the same group, *P* < 0.05 after Bonferroni correction. ^c^Compared with the 12h value in the same group, *P* < 0.05 after Bonferroni correction

*HFNC* High-flow nasal cannula oxygen therapy; *NIV* Non-invasive ventilation; *PaCO*_*2*_ Partial pressure of arterial carbon dioxide; *PaO*_*2*_ Partial pressure of arterial oxygen; *FiO*_*2*_ Fraction of inspiration oxygen

Arterial blood gas analyses showed that, the pH and the PaO_2_/FiO_2_ values were all significantly elevated at one hour, 12 h and 48 h after initiating treatment, but there was no significant difference between the two groups at each time point. The PaCO_2_ in both groups was decreased at one hour and 12 h after initiating treatment. At 48 h, the PaCO_2_ in the NIV group continued to decrease and was significantly lower than in the HFNC group [53 (49–59) mmHg vs 57 (52–60) mmHg, *P* = 0.043, see Table 4].

**Fig. 3:**
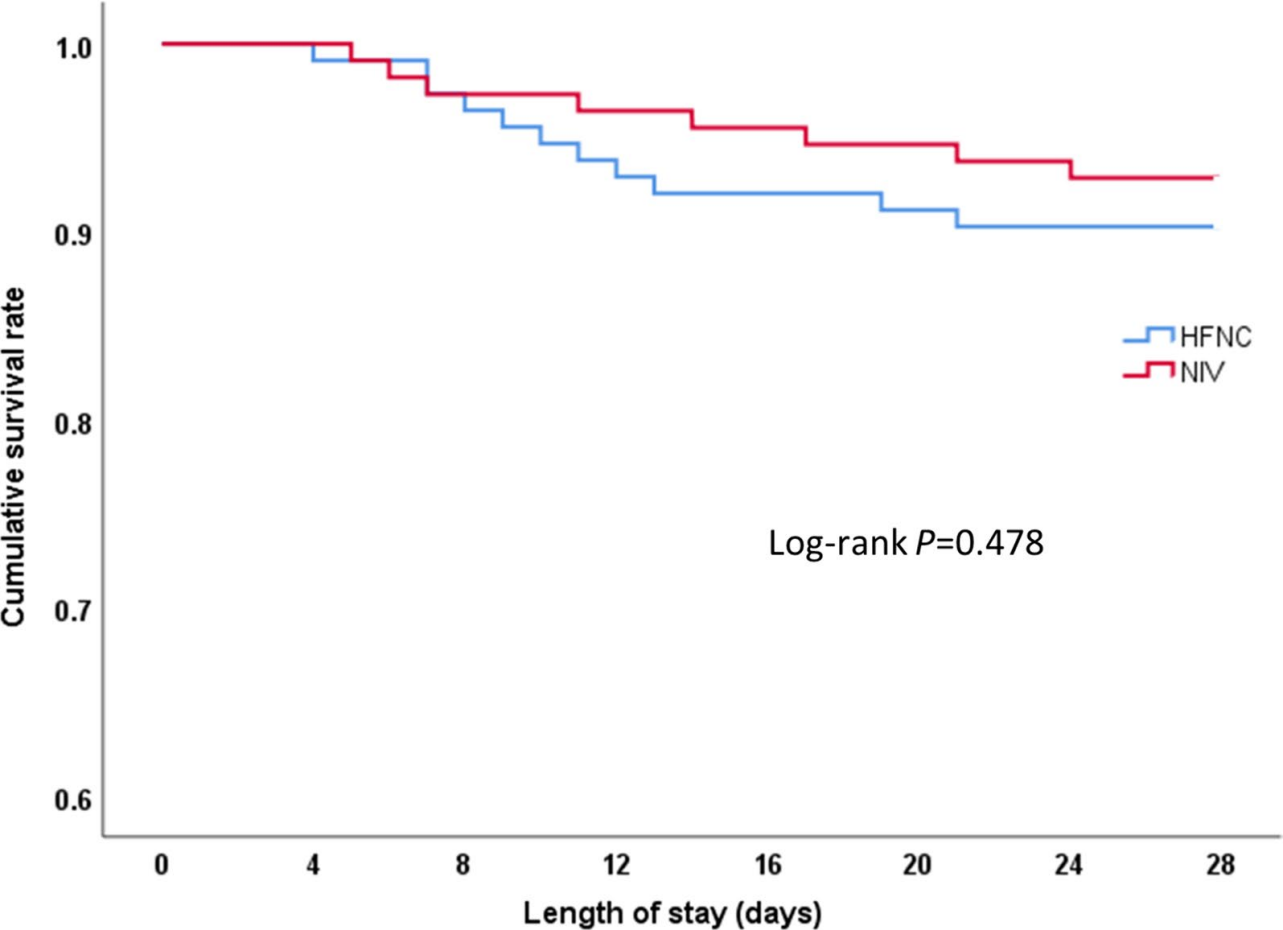
Kaplan–Meier curve analysis for cumulative survival rate. HFNC: High-flow nasal cannula oxygen therapy; NIV: Non-invasive ventilation

There were no significant differences in ICU or hospital total lengths of stay between the two groups (all *P* < 0.050, see Table 3). The 28-day mortality in the HFNC group was 9.7%, which was not significantly different from the 7.1% rate in the NIV group (Log Rank test 0.504, *P* = 0.478, see Fig. 3).

### Other characteristics in the HFNC and NIV groups

There were no significant differences in the total duration of respiratory support (HFNC or NIV), dyspnea scores or treatment intolerance between the two groups (all *P* > 0.050, see Table 5). In the analysis of respiratory support duration in the first five days, the support duration in the HFNC group was significantly longer than that in the NIV group during the first two days (*P* < 0.05), but there was no significant difference between the two groups during the last three days. The number of daily airway care interventions and the incidence of nasofacial skin breakdown were significantly lower in the HFNC group than in the NIV group (all *P* < 0.050, see Table 5). The comfort score in the HFNC group was significantly higher than in the NIV group [7 (6–8) vs 6 (5–7), *P* < 0.001].

**Table 5:** Other characteristics in the HFNC and NIV groups

	HFNC (n = 113)	NIV (n = 112)	*P* value
Duration of HFNC or NIV (hours)	85.9 ± 30.5	78.7 ± 33.8	0.312
Day 1 (hours)	13.2 ± 4.5	9.9 ± 3.5	< 0.001
Day 2 (hours)	16.3 ± 4.1	12.8 ± 3.9	0.005
Day 3 (hours)	15.9 ± 6.4	14.8 ± 8.6	0.068
Day 4 (hours)	14.9 ± 6.5	13.6 ± 8.5	0.075
Day 5 (hours)	12.8 ± 4.9	11.4 ± 7.9	0.125
Dyspnea score	2 (2–4)	2 (1–3)	0.085
Airway care interventions, per day	5 (3–7)	8 (6–10)	< 0.001
Comfort score	7 (6–8)	6 (5–7)	< 0.001
Treatment intolerance	1 (0.9)	5 (4.5)	0.210
Nasal facial skin breakdown, n (%)	3 (2.7)	10 (8.9)	0.044

*HFNC* High-flow nasal cannula oxygen therapy; *NIV* Non-invasive ventilation; *ICU* Intensive care unit

## Discussion

This non-inferiority RCT showed that HFNC was not shown to be non-inferior to NIV for preventing treatment failure for AECOPD patients with moderate hypercapnic ARF in both an intention-to-treat analysis or in a per-protocol analysis. Both analyses suggested that NIV was superior to HFNC in terms of treatment failure rates. The rate of endotracheal intubation in the HFNC group was higher than that in the NIV group. NIV was better than HFNC in reducing PaCO_2_ at 48 h after initiating respiratory treatment. HFNC had better results compared to NIV in both patient-reported comfort and in the number of nursing airway interventions.

Increasing numbers of studies have explored the efficacy of HFNC for COPD patients. Initially, evidence to support the use of HFNC was mainly limited to stable hypercapnic COPD. Several studies have shown that HFNC can improve exercise tolerance and quality of life in stable COPD patients, while relieving carbon dioxide retention, decreasing RR, and reducing acute attacks and hospitalization times [14–16].

Research focusing on the application of HFNC to AECOPD is now starting to emerge. A recent RCT showed that, in AECOPD patients with acute compensatory hypercapnic respiratory failure (pH ≥ 7.35, PaO_2_ < 60 mmHg, and PaCO_2_ > 45 mmHg), HFNC significantly reduced the need for invasive ventilation or NIV compared with conventional oxygen therapy, and PaCO_2_ after 24 h of treatment was lower in the HFNC group [17]. However, another multicenter RCT by Xia et al. found that HFNC could not reduce the need for intubation compared with conventional oxygen therapy among AECOPD patients with pH ≥ 7.35 and PaCO_2_ > 45 mmHg, and the authors suggested to explore the effect of HFNC on AECOPD patients with a pH < 7.35 [18].

Few studies comparing HFNC to NIV in AECOPD as the initial treatment choice have yet been published. In an RCT involving 72 AECOPD patients with a PaCO_2_ > 50 mmHg, HFNC was reported to have lower PaCO_2_, higher oxygenation and comfort scores than NIV, and showed no significant difference in intubation rates between HFNC and NIV [19]. In our study, however, the treatment failure and intubation rates of HFNC were significantly higher than in NIV. Oxygenation was similar between the two groups, though HFNC had better patient comfort scores than NIV. The aggravation of carbon dioxide retention was the most common reason for treatment failure in the HFNC group in our study.

Several studies have observed the effect of HFNC on reducing PaCO_2_ in patients with AECOPD. A multicenter observational case series study showed that HFNC treatment for one hour can significantly decrease the RR and PaCO_2_ when trialed among 40 COPD patients with hypercapnic ARF [20]. A randomized study by Pilcher et al. found that half an hour of HFNC application decreased PaCO_2_ by 1.4 mmHg and RR by 2 breaths/min compared with conventional oxygen therapy [21]. A cross-over randomized study showed that both HFNC and NIV treatment for one hour can improve respiratory acidosis and relieve dyspnea in AECOPD patients, and HFNC was more effective than NIV for improving both oxygenation and RR [22].

These studies demonstrated that the ability of HFNC to reduce PaCO_2_ in the short term was better than conventional oxygen therapy, and appeared to be non-inferior to NIV. Regarding the short term results, we found similar outcomes in our study, in which the values of PaCO_2_ between HFNC and NIV were similar at one hour and 12 h. However, PaCO_2_ in the NIV group continued to decrease at 48 h and was lower than in the HFNC group in our study. In the RCT by Cortegiani et al. 32% of patients in the HFNC group received NIV within 6 h, though HFNC and NIV had similar effects on reducing PaCO_2_ after two hours treatment in AECOPD patients with a pH 7.25–7.35 [23]. Although HFNC was found to be superior to NIV in reducing PaCO_2_ at discharge in an RCT that enrolled 40 patients with hypercapnic ARF (62.5% COPD patients) [24], we still suggest that the effect of HFNC on reducing PaCO_2_ in AECOPD patients should be treated with caution, especially after several days of initial treatment.

Low levels of positive airway pressure, the wash-out effect of exhaled gas in the upper airways and reduced physiological dead-space are the main physiological bases for HFNC to decrease PaCO_2_ [25]. During the initial stage of treatment, these effects can effectively remove carbon dioxide. However, the pulmonary function of AECOPD patients with hypercapnic ARF is usually poor, while the inducing factors such as infection have most likely not yet been controlled during the initial treatment stage, so the patient’s ill condition may not yet have reached its peak. With the passage of time, powerful respiratory support may be needed to control respiratory acidosis. Compared with HFNC, NIV has adjustable positive end-expiratory pressure and extra pressure support abilities, which can ensure a patient’s minute ventilation volume more effectively than HFNC. This may be the main reason why the PaCO_2_ in the NIV group was lower than that in the HFNC group after 48 h of treatment in our study.

Although several meta-analyses have shown that the efficacy of HFNC in hypercapnic ARF was comparable to that of NIV [26, 27], the current body of evidence may not be enough to draw a clear conclusion given study heterogeneity [28]. Non-inferior RCTs comparing HFNC and NIV in AECOPD patients with larger sample sizes have been rarely reported until now. This RCT suggested that the treatment failure rate of HFNC is higher than that of NIV. In other studies, the effectiveness of HFNC in AECOPD with hypercapnic ARF has also been questioned. A retrospective cohort study based on the MIMIC-IV database showed that the length of ICU stay in the HFNC group was significantly longer than that of the NIV group, while the 48-h intubation rate, 28-day intubation rate and 28-day mortality rate in the HFNC group were all higher than in the NIV group [29]. Additionally, it could be that the prolonged length of hospital stay in the HFNC group may be due to delayed or reduced escalation to NIV treatment [30].

As in this study, the better comfort of HFNC over NIV has been confirmed by many studies. This may be the reason why the treatment duration in the HFNC group was longer than in the NIV group on the first and second day. However, the tolerance of NIV can be improved by staff training, patient education and quality improvement [31]. Therefore, there was no difference in the duration of respiratory support between the two groups from the third day to the fifth day. The total endotracheal intubation rate in this study was 9.8%, which was lower than previous studies [1]. This may be related to our center’s many years of experience in using NIV and HFNC, as well as a clear quality improvement framework for both NIV and HFNC, including rapid identification, fast commencement, staff training, escalation protocols and close monitoring in our clinical practice. Moreover, a low endotracheal intubation rate of about 3% was also recently reported in some studies [19, 23].

Our study has some limitations. First, this was a single-center study and multi-center trials would be needed to confirm the external validity of our findings. Second, blinding was not possible to attending physicians or patients due to the treatments utilizing clearly different devices. However, the data analyst was blinded to the study groups and investigators were excluded from clinical decisions to help reduce bias. Third, the initial gas flow rate of HFNC in this study was set to 40 L/min. Whether other flow rates would have a better effect on carbon dioxide removal is worth exploring in future studies. Finally, the switch between HFNC and NIV in this study was decided by each patient’s attending physician, which was influenced by many factors and does introduce a certain subjectivity, despite the study having certain respiratory markers to assist in guiding care. Nevertheless, this study may better reflect the pragmatic application of NIV or HFNC in actual clinical practice.

## Conclusions

In AECOPD patients with moderate hypercapnic ARF, HFNC was not shown to be non-inferior to NIV and resulted in a higher incidence of treatment failure than NIV when used as initial respiratory support.

## Data Availability

The datasets used and analyzed during the current study are available from the corresponding author in response to reasonable requests.

## Abbreviations

COPD: Chronic obstructive pulmonary disease
AECOPD: Acute exacerbations of chronic obstructive pulmonary disease
ARF: Acute respiratory failure
NIV: Non-invasive ventilation
HFNC: High-flow nasal cannula oxygen therapy
RCT: Randomized controlled trial
ICU: Intensive care unit
FiO_2_: Fraction of inspiration oxygen
SPO_2_: Pulse oxygen saturation
APACHE II: Acute physiological and chronic health status score II
SAPS II: Simplified acute physiology score II
PaCO_2_: Partial pressure of arterial carbon dioxide
PaO_2_: Partial pressure of arterial oxygen
RR: Respiratory rate
